# Discovery and Characterization of a New Crustin Antimicrobial Peptide from *Amphibalanus amphitrite*

**DOI:** 10.3390/pharmaceutics14020413

**Published:** 2022-02-14

**Authors:** Wei Zhang, Xiaohang Xu, Jun Zhang, Ting Ye, Qiao Zhou, Ying Xu, Wenyi Li, Zhangli Hu, Chenjing Shang

**Affiliations:** 1 Shenzhen Key Laboratory of Marine Bioresource and Eco-Environmental Science, Shenzhen Engineering Laboratory for Marine Algal Biotechnology, Guangdong Provincial Key Laboratory for Plant Epigenetics, College of Life Sciences and Oceanography, Shenzhen University, Shenzhen 518060, China; zhangwei1994@szu.edu.cn (W.Z.); 2019305001@email.szu.edu.cn (X.X.); 2100252001@email.szu.edu.cn (T.Y.); zhouqiao@szu.edu.cn (Q.Z.); boxuying@szu.edu.cn (Y.X.); huzl@szu.edu.cn (Z.H.); 2 College of Physics and Optoelectronic Engineering, Shenzhen University, Shenzhen 518060, China; 3 Institute of Systems and Physical Biology, Shenzhen Bay Laboratory, Shenzhen 518055, China; zhangjun@szbl.ac.cn; 4 The Bio21 Institute of Molecular Science and Biotechnology, Faculty of Medicine, Dentistry and Health Sciences, The University of Melbourne, Melbourne, VIC 3010, Australia

**Keywords:** antimicrobial peptides, crustin, membrane active, *Amphibalanus amphitrite*, antibiotic, antibiofilm

## Abstract

Crustins are an antimicrobial peptide (AMP) family that plays an important role in innate immunity in crustaceans. It is important to discover new AMPs from natural sources to expand the current database. Here, we identified and characterized a new crustin family member, named *Aa*Crus1, from *Amphibalanus amphitrite*. *Aa*Crus1 shares high identity (48.10%) with *PvCrus*, a Type I crustin of *Penaeus vannamei* that possesses a whey acidic protein (WAP) domain. *Aa*Crus1 contains 237 amino acids and eight cysteine residues forming conserved ‘four-disulfide core’ structure. Our recombinant *Aa*Crus1 (r*Aa*Crus 1) could inhibit the growth of two Gram-positive bacteria (*Staphylococcus aureus*, *Bacillus* sp. T2) and four Gram-negative bacteria (*Vibrio parahaemolyticus*, *Vibrio harveyi*, *Vibrio anguillarum*, *Vibrio alginolyticus*) with a minimum inhibitory concentration of 3.5–28 μM. It can further induce agglutination of both Gram-positive and Gram-negative bacteria. r*Aa*Crus1 can bind to bacteria and damage bacterial cell membranes. Furthermore, r*Aa*Crus1 disrupted biofilm development of *S. aureus* and *V. parahaemolyticus*. Our discovery and characterization of this new crustin can be further optimized as a good alternative to antibiotics.

## 1. Introduction

The advent of antibiotics effectively treats diseases caused by bacterial infections. Furthermore, antibiotics are used in aquaculture to treat pathogenic infections in aquaculture animals. *Vibrio* sp. infection is one of the main diseases hindering the development of aquaculture, and some *Vibrio* sp. can cause serious foodborne illness in humans and animals [1]. However, the overdosed antibiotics and global interconnectedness has accelerated the emergence of antibiotic-resistant strains and ecological pollution. The World Health Organization has urgently called upon scientists to find novel effective alternatives to antibiotics. Drug-resistant *Vibrio parahaemolyticus* strains have been detected in China, South Korea and other places [1]. Antimicrobial peptides (AMPs), also known as host defense peptides, are a class of short peptides with biological activity that are widely present in nature [2,3]. As an important part of the biological innate immune system, AMPs have broad-spectrum antibacterial properties, which have a mild inhibitory or killing effect on Gram-negative bacteria, Gram-positive bacteria, fungi, viruses and parasites [4]. AMPs are less likely to increase the resistance of bacteria and are excellent alternatives to antibiotics [2,5,6].

Crustaceans are ancient animals with a large number and variety which are widely distributed in the ocean, freshwater ponds and lakes. Crustaceans mainly use humoral immunity, cellular immunity and other innate immune methods to combat pathogenic microorganisms. As part of their immune system, AMPs from crustaceans display remarkable diversity in their structural and genetic composition, which can serve as future templates for novel antimicrobial agents [7,8]. Since the first crustacean AMP was discovered in *Carcinus maenas* in 1996 [9], there are around 70 crustacean AMPs now recorded in the ADP database (http://aps.unmc.edu/AP/, accessed on 1 December 2021). Due to its species diversity, more new crustacean antibacterial peptides are being discovered annually [10,11].

**Figure 1:**
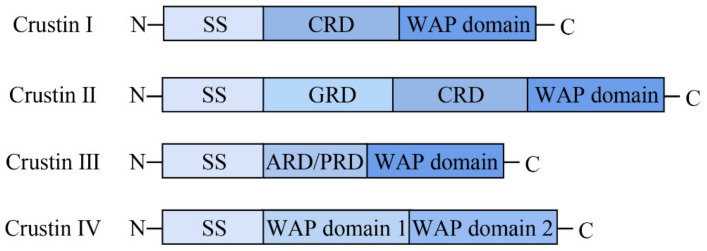
Schematic representation (not to scale) of the structural organization of the four crustin Types (I to IV) found in crustaceans.

Crustins, discovered in crustaceans, are generally cationic AMPs with 6–22 kDa and contain twelve conserved cysteine residues, eight of which comprise a typical whey acid protein (WAP) domain [12]. The WAP domain consist of a four-disulfide bond core arrangement at the C-terminus and is potentially associated with multiple functions, such as antimicrobial activity and proteinase inhibition [13,14]. The WAP domain at the C-terminus is very conservative, but the length and amino acid sequence at the N-terminus are highly variable [13,14]. As shown in Figure 1, according to the sequence difference in N-terminal, crustins were divided initially into four members [10,11]. The N-terminal has a cysteine-rich region that is to be found in type I crustin [15]. Type II crustins have a glycine-rich region between the cysteine-rich region and the signal peptide region [12]. Type III crustins have no cysteine-rich region and glycine-rich region, only a proline-rich region and WAP domain [16]. Type IV crustins have two WAP domains [17]. Though the four types of crustins all show good antimicrobial activity, different types of crustins with various structures cause differences in antibacterial effects. Some studies found that crustins may inhibit bacterial growth through protease inhibitory or cell membrane damage [11,14].

It is generally believed that the WAP domain of the four disulfide bonds in crustins is the key area for the antibacterial function [12,14]. The WAP domain of a crustin (*Re*Crus1) found in *Rimicaris exoculata* has an independent and different division of labor from other regions. The other regions are responsible for the binding of crustin to bacteria, and the WAP domain is responsible for killing bacteria [14]. Since AMPs with glycine and lysine rich are highly selective for Gram-negative bacteria [18], the glycine-rich region may be one of the reasons for the antibacterial effect of type II crustin. Some crustins have agglutinating activity by cross-linking with bacterial surface components to form a crystal lattice [19,20,21,22]. However, there are very few studies on crustins from non-decapod crustaceans [12,19].

*A. amphitrite* (Crustacea: Cirripedia) is a small striped barnacle commonly found in harbors and on ship hulls around the world. The microbial community and the development of barnacles influence each other, and the microbial community can affect the development of barnacles, promote or prevent barnacle larvae’s settlement and colonization [20,21]. Barnacles secrete lectin, serotonin and other substances to transform the microbial community around the microorganisms, and at the same time, need to use innate immunity to resist the invasion of pathogenic microorganisms [20]. Therefore, there may be AMPs with excellent antibacterial effects in barnacles. In order to find novel crustins in *A. amphitrite*, we constructed a crustin protein sequence database and compared the protein sequence of *A. amphitrite* in the NCBI database, thus identifying a new crustin member of the crustins family. With recombinant expression of the new crustin in *Escherichia coli*, we obtained a pure crustin protein without purification tag, named recombinant *Aa*Crus1 (r*Aa*Crus1). To further explore the *Aa*Crus1 application in aquaculture industry, we evaluated its antibacterial activity and investigated the possible antibacterial mechanism against multiple *Vibrio* pathogens in aquaculture, including *Vibrio parahaemolyticus*, *Vibrio harveyi*, *Vibrio anguillarum* and *Vibrio alginolyticus*.

## 2. Materials and Methods

### 2.1. Bacterial Strains and Culture Conditions

The Gram-positive bacteria (*Staphylococcus aureus*, *Bacillus* sp. T2) and the Gram-negative bacteria (*Vibrio parahaemolyticus*, *Vibrio harveyi*, *Vibrio anguillarum*, *Vibrio alginolyticus*) were a gift from Professor Chaogang Wang [21,22]. They were originally glycerol bacteria and were stored at –80 °C. Before the experiment, the strains (glycerol bacteria) were cultured with Luria-Bertan (LB) medium shaken at 200 rpm, 37 °C overnight (usually about 10 h).

### 2.2. Prediction and Identification of Crustin Sequences

*A. amphitrite* genome information (NCBI access number: GCF_019059575.1) and 90 crustin sequences of *Penaeus vannamei* (49), *Penaeus monodon* (23) and *Portunus trituberculatus* (18) were obtained from NCBI. A local database was built using crustin sequences from other species by makeblastdb. We subsequently employed BLASTP (e-value: 1 × 10^−5^) to identify putative crustin sequences from *A. amphitrite* using the local database. Sequence homology searches were performed by the Protein BLAST algorithm in the NCBI (https://blast.ncbi.nlm.nih.gov/Blast.cgi, accessed on 1 December 2021). Signal peptide was identified by SignalP 5.0 [23]. Multiple alignments of amino acid sequences were built with ClustalW (https://www.genome.jp/tools-bin/clustalw, accessed on 1 December 2021), and the output pattern was visualized by DNAMAN 8 (Lynnon Biosoft, San Ramon, CA, USA). The neighbor-joining phylogenetic tree was performed by MEGA-X with 1000 bootstraps as assess reliability (Mega Limited, Auckland, New Zealand). ITOL6.3.2 was used to modify the evolutionary tree [24]. The full-length atomic model of r*Aa*Crus1 was constructed with iterative template-based fragment assembly simulations using I-TASSER [25]. Protein physicochemical properties were predicted by ProtParam (https://web.expasy.org/protparam/, accessed on 1 December 2021).

### 2.3. Expression and Purification of Recombinant AaCrus1 (rAaCrus1)

The mature peptide of *Aa*Crus1 was fused to the C-terminus of the His-SUMO tag, and prokaryotic expression was performed in *E. coli* BL21 (DE3) (TransGen Biotech, Beijing, China). The nucleotides in *Aa*Crus1 (NCBI access number: VIIS01000966.1) were optimized according to the codon preference of *E. coli*, and BamHI and XhoI restriction sites were added at both ends of the sequence. The nucleotide sequence of the mature peptide of *Aa*Crus1 is 726 bp. The gene was synthesized by the chemical synthesis method by General Biosystems (Anhui) Company (Chuzhou, China), and the synthesized DNA was ligated with pSmartI-SUMO vector through BamHI and XhoI restriction sites, and pSmartI-SUMO was provided by General Biosystems (Anhui) Company (Chuzhou, Anhui, China). The resulting recombinant plasmid was named pSmartI-SUMO-*Aa*Crus1 (Supplementary Figure S1). pSmart-SUMO-*Aa*Crus1 plasmid size is 6274 bp. *Aa*Crus1EF: 5′-TTA AGA TTC TTG TAC GAC GG-3′ and *Aa*Crus1ER: 5′-TGC TAG TTA TTG CTC AGC GG-3′ were primers. The PCR was performed at 95 °C for 3 min, with 35 cycles of 95 °C, 30 s, 51 °C, 30 s, 72 °C, 1 min and 72 °C, 5 min. General Biosystems (Anhui) Company delivers pSmartI-SUMO-*Aa*Crus1 in the form of lyophilized powder. pSmartI-SUMO-*Aa*Crus1 was transformed into *E. coli* BL21 (DE3) by heat shock method, and positive single clones were screened with solid LB medium contain kanamycin (50 µg/mL). Positive single clones confirmed by PCR and sequencing were used for recombinant protein production [26]. The positive signal clones were inoculated in LB medium containing kanamycin, and the bacterial solution was shaken at 200 r/min at 37 °C for overnight incubation. The next day, the bacterial liquid cultured overnight was added to fresh LB medium at a volume ratio of 1:100, the bacterial liquid was shaken at 37 °C at 200 r/min, and the bacterial liquid was cultured to OD600 = 0.6. Recombinant protein expression was induced by the addition of isopropyl-β-d-thiogalactoside (IPTG) to an ultimate concentration of 0.8 mM at 37 °C for 6 h. The cells were broken by ultrasonic means for 40 min (power: 195 W, ultrasonic for 2 s, pause for 4 s) and centrifuged at 4 °C for 30 min at 10,000 r/min to separate the supernatant and precipitate. The crude protein in the uninduced cells and the induced cells was extracted for 12% SDS-PAGE, and the crude protein in the induced cells was subjected to SDS-PAGE and Western Blot. The His-SUMO-*Aa*Crus1 protein from the supernatant was isolated and purified by Ni-column affinity chromatography. The His-Sumo-*Aa*Crus1 protein was dialyzed against 1× PBS for 24 h at 4 °C. His-SUMO-*Aa*Crus1 protein was detected by sodium dodecyl sulfate-polyacrylamide gel electrophoresis (SDS-PAGE). For Western Blot, proteins were transferred onto a polyvinylidene fluoride membrane (PVDF), which was blocked with 5% skim milk in 1× TBST. The blot was incubated with His-Tag Mouse Monoclonal Antibody (1:1000, Beyotime, Shanghai, China) and a horseradish peroxidase-conjugated goat anti-mouse secondary antibody (1:2000, Beyotime, Shanghai, China), respectively. Detection was performed with the BeyoECL Plus (Beyotime, Shanghai, China) according to the manufacturer’s instructions. In order to completely remove the SUMO tag, 1 U SUMO (purchased from General Biosystems (Anhui) Company (Chuzhou, China)) enzyme was used at 4 °C for 6 h. The SUMO tag, which contained His tag, was intercepted by Ni-column affinity chromatography, and the unattached liquid was r*Aa*Crus1 protein solution without tag. The purified r*Aa*Crus1 protein was analyzed by SDS-PAGE. The protein concentration was determined using the BSA Protein Assay Kit (Beyotime, Shanghai, China) according to the manufacturer’s instruction. Finally, the protein solution was separated and lyophilized to obtain protein powder stored at −80 °C.

### 2.4. Identify rAaCrus1 by Liquid Chromatograph Mass Spectrometer (LC-MS)

The r*Aa*Crus1 protein band obtained by SDS-PAGE gel electrophoresis was cut and placed in a 1.5 mL Eppendorf centrifuge tube, 50% volume fraction of acetonitrile was added, and the color was decolorized overnight at 37 °C with 200 r/min shaking. Using pure acetonitrile to make the colloid white and hard, the supernatant was discarded, trypsin digestion added, and then the covering solution (ammonium bicarbonate) was added in a 37 °C water bath for 16 h. After digestion by trypsin Eppendorf centrifuge tube, the solution was transferred to a new 1.5 mL, the extract added (water and anhydrous acetonitrile mixed in a volume ratio of 1:4, add 0.5% of the total volume of formic acid), sonicated for 10 min, centrifuged, and then the extract was vacuum dried for 4 h to obtain protein powder. The protein powder was dissolved with the sample solution (water and anhydrous acetonitrile in a volume ratio of 1:49, then add 0.5% formic acid) to dissolve the protein powder, vortexed and shaken 13,000× *g* at room temperature for 15 min, and finally, the LC-MS (TRIPLETOF 5600+, AB SCIEX, Singapore) was used to identify r*Aa*Crus1.

### 2.5. Antimicrobial Activity of the rAaCrus1

In order to find as much novel potential r*Aa*Crus1 as possible, we thus performed a modified CLSI microtiter plate assay to test the minimum inhibition concentration (MIC) with 10^4^ CFU mL^−1^. The bacteria were cultured with the method described above until OD_600_ reached 0.4, and then diluted into 10^4^ CFU mL^−1^ with Mueller–Hinton Broth medium. r*Aa*Crus1 were dissolved with 1× PBS, and 20 μL peptide was mixed with 80 μL diluted strains in a 96 well plate. A serious final concentration of the peptide (56 μM, 28 μM, 14 μM, 7 μM, 3.5 μM, 1.75 μM, 0.875 μM) was used for MIC, and 1× PBS was the negative control. The plate was incubated at 37 °C for 18 h and OD_600_ was checked at 0 h and 18 h. The lowest concentration at which no growth of the indicator was observed was considered as the MIC of the peptide. The assay was performed with three times biological replicates and three times technical replicates.

### 2.6. Molecular Dynamics (MD) Simulations

Given the importance of peptide–membrane interaction under computational simulations [27,28]. A series of MD simulations were performed to study the interactions between *Aa*Crus1 and membrane. *Aa*Crus1 was described with the GROMOS 54a7 force field [29]. The membrane was modelled with 128 dipalmitoylphosphatidylcholine (DPPC) molecules in a united-atom force field by Berger et al. [30]. The solvent was represented by SPC water [31]. Two systems were simulated: *Aa*Crus1 along and *Aa*Crus1 membrane. A solvent box of 7.56 × 7.56 × 7.56 nm^3^ and 6.42 × 6.44 × 12.00 nm^3^ was built for the former and latter systems, respectively. In both systems, ions were added to maintain the neutral charge.

All MD simulations were carried out with GROMACS 2019.3 [32]. The cutoff for the short-range part of the Lennard–Jones and Coulomb interaction was set to 1.5 nm. The particle mesh Ewald algorithm [33,34] was used to compute the long-range part of the Coulomb interaction (with a grid space of 0.12 nm and a spline of order 4). The temperature was controlled at 323 K (above the phase transition temperature of DPPC) using the Nosé–Hoover algorithm [35,36] with the time constant of 0.5 ps. The pressure was maintained at 1 bar with the Parrinello–Rahman algorithm [37] in a semi-isotropic way where the time constant and compressibility was set to 5 ps and 5 × 10^−5^ (kJ·mol^−1^·nm^−3^)^−1^, respectively. All bonds were fixed using the LINCS algorithm [38], thus a time step of 2 fs could be applied. For each system, energy was first minimized, then was equilibrated under NVT condition for 200 ns, followed by an NPT simulation for 400 ns. The last 200 ns of trajectories were used for analysis.

### 2.7. Microorganism-Binding Assay

Western Blot experiment was used to detect the binding activity of r*Aa*Crus1 to bacteria. Briefly, the microorganisms (1 × 10^8^ CFU) in a 1.5 mL centrifuge tube were incubated in 200 μL of His-SUMO-*Aa*Crus1(20 μM) by gentle rotation for 30 min at room temperature. The cells were collected and washed three times with 1× Tris Buffered Saline (TBS) and then resuspended. After the centrifugation at 10,000 rpm for 5 min, the bacteria and supernatant were directly loaded on SDS-PAGE for analysis, respectively. r*Aa*Crus1 was the positive control. Finally, proteins were transferred onto a polyvinylidene fluoride membrane (PVDF), which was blocked with 5% skim milk in 1× TBST. The blot was incubated with His-Tag Mouse Monoclonal Antibody (1:1000, Beyotime, Shanghai, China) and a horseradish peroxidase-conjugated goat anti-mouse secondary antibody (1:2000, Beyotime, Shanghai, China), respectively. Detection was completed with the BeyoECL Plus (Beyotime, Shanghai, China), according to the manufacturer’s instructions.

### 2.8. Agglutinating Assay

To understand the function mechanism of r*Aa*Crus1, six microorganisms described previously were selected for the agglutination assay. Briefly, six microorganisms were cultured at mid-logarithmic phase, centrifuged at 5000× *g* for 5 min, washed with 1× TBS four times and resuspended in 1× TBS to 10^8^ CFU·mL^−1^. The microorganism suspension (30 μL) was incubated with a series of dilutions of r*Aa*Crus1 in the range of 0.875–56 μM in 1× TBS (30 μL) with the absence or presence of 5 mM CaCl_2_ at 37 °C for 1 h. Compared to the negative control (400 μg/mL BSA), the minimal agglutinating concentration (MAC) in this research was identified as the lowest protein concentration harvesting apparent agglutination function. Agglutination was then observed under a Leica DMi1 Microscope (Leica, Wetzlar, Germany). All treatments were performed with three times biological replicates and three times technical replicates.

### 2.9. Electron Microscopy

The electron microscope was carried out with reference to previous studies [14]. *S. aureus*, *V. anguillarum* and *V. alginolyticus* were cultured in Luria–Bertan medium to mid-log phase. Then, the bacteria were collected and resuspended to 10^6^ CFU/mL in 1× PBS. The bacteria were cultured at 2 × MIC r*Aa*Crus1 concentration for 2 h on round coverslip in 24-well plates. After treatment, the cells were fixed with 5% glutaraldehyde in PBS (pH 7.4) for 2 h at 4 °C, followed by washing three times with 1× PBS. The bacteria treated with 1× PBS was the control. The bacteria were dehydrated in a series of concentrations of ethanol (30, 50, 70, 80, 90 and 100%) for 10 min at 4 °C and incubated with isoamyl acetate for 10 min [14]. The cells were sputter-coated with platinum (MC1000, Hitachi, Tokyo, Japan) after critically point-dried (Hitachi-HCP, Hitachi, Tokyo, Japan), and examined with scan electron microscopy (SEM) (APREO S, Thermo, Eindhoven, The Netherlands).

### 2.10. PI Staining Assay

*S. aureus*, *V. anguillarum* and *V. alginolyticus* were cultured as described above at their 2 × MIC concentration for 2 h. Then, the samples were stained with a PI staining kit (Sangon, Shanghai, China) according to the manufacturer’s instructions. The cells were observed with a fluorescence microscope (BX51, Olympus, Tokyo, Japan).

### 2.11. Biofilm Inhibition

*S. aureus* and *V. parahaemolyticus* were cultured according to the method described above. After the test strain was cultured at OD600 of 0.4, it was transferred to a 96-well plate treated with TC, and 200 μL of bacterial liquid was added to each well and cultured for 12 h. The bacterial solution was aspirated and the plate was washed with sterile PBS (pH 7.5). Then, r*Aa*Crus1 solution (10–50 μM) was added to the wells and incubated at 37 °C for 24 h. After discarding the r*Aa*Crus1 solution, the 96-well plates were washed with sterile 1× PBS (pH 7.5), then 100 μL 0.2% crystal violet staining solution (*w*/*v*) was added to each well and washed with sterile H_2_O after staining for 15 min. Once dry, 100 μL of absolute ethanol was added to each well, and the absorbance at a wavelength of 570 nm was measured. For microscopic analysis, the strains to be tested were cultured to an OD600 of 0.4, then transferred to a 24-well plate, where round coverslips were placed, and 200 μL of bacterial liquid was added to each well and cultured for 12 h. The round coverslips were then removed and washed with sterile PBS (pH 7.5). Then, round coverslips with biofilm were placed in a new 24-well plate, and r*Aa*Crus1 solution (10–50 μM) was added to the 24-well plate so that it covered the round coverslips. Round coverslips were collected after incubating the 24-well plate at 37 °C for 24 h. After crystal violet staining, stained glass slides were placed on glass slides with the biofilm facing up and observed under a light microscope at magnification of 40× [39].

### 2.12. Statistical Analysis

GraphPad Prism 7.0 (GraphPad, San Diego, CA, USA) was selected for statistical analysis. Statistical significance was determined with one-way analysis of variance (ANOVA). All data are presented as mean ± SD. *p* value < 0.05 was considered statistically significant.

## 3. Results

### 3.1. Sequence and Structure Characterization of AaCrus1

The amino acid sequence of *Aa*Crus1 (NCBI access number: KAF0303230,1) contains 257 residues with a calculated molecular weight of 26.63 kDa and a predicted pI of 8.47 by ProtParam. The CDS sequence encoding *Aa*Crus1 is 774 bp. The nucleotide sequence of the mature peptide of *Aa*Crus1 is 726 bp.

**Figure 2:**
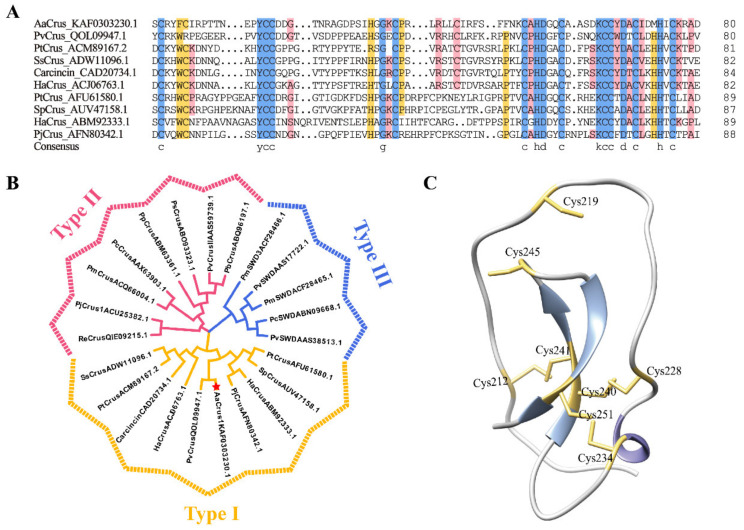
Sequence alignments and phylogenetic and structural analysis of *Aa*Crus1. (**A**) Alignment of *Aa*Crus1 WAP domain and cysteine-rich domain with Type I crustins (shown in Supplementary Table S2). The consensus residues are shaded red; the residues that are ≥75% identical among the aligned sequences are shaded blue. The consensus residues are shaded red; the residues that are ≥75% identical among the aligned sequences are shaded blue. (**B**) Phylogenetic analysis of *Aa*Crus1 homologues. The phylogenetic tree was constructed with MEGA-X using the neighbor-joining method. Numbers beside the internal branches indicate bootstrap values based on 1000 replications. The GenBank accession numbers of the crustins are indicated after crustins’ names. The crustin information used to construct the evolutionary tree is in Supplementary Table S3. (**C**) The predicted structure of Crus1 was built using I-TASSER. The cysteines in the WAP domain are shown in yellow, β-pleated sheet is shown in blue and α-helix is shown in purple.

*Aa*Crus1 possesses a signal peptide in the N-terminus (residues 1 to 20) and a WAP domain in the C-terminus, in which a ‘four-disulfide core’ structure can be formed by Cys212–Cys241, Cys219–Cys245, Cys228–Cys240 and Cys234–Cys251. Protein BLAST showed that *Aa*Crus1 shares the highest identity (48.10%) with *Pv*Crus, a Type I crustin of *Penaeus vannamei* (GenBank accession No. QOL09947.1). Sequence alignment between *Aa*Crus1 and representative type I crustins showed that they both have eight cysteines that form a tetradisulfide core structure on the WAP domain (Figure 2A). In addition, four cysteines before WAP domain (corresponding to Cys180, Cys184, Cys198 and Cys199 in *Aa*Crus1) were also conserved among the Type I crustins (Figure 2A). Compared with other type I crustins, there is a longer sequence between the signal peptide region and the cysteine-rich region. Phylogenetic analysis showed that *Aa*Crus1 was grouped into the clade of type I crustins (Figure 2B). The predicted protein structure of *Aa*Crus1 contains random coils, α-helix and β-pleated sheet (Figure 2C).

### 3.2. Recombinant Expression and Purification

**Figure 3:**
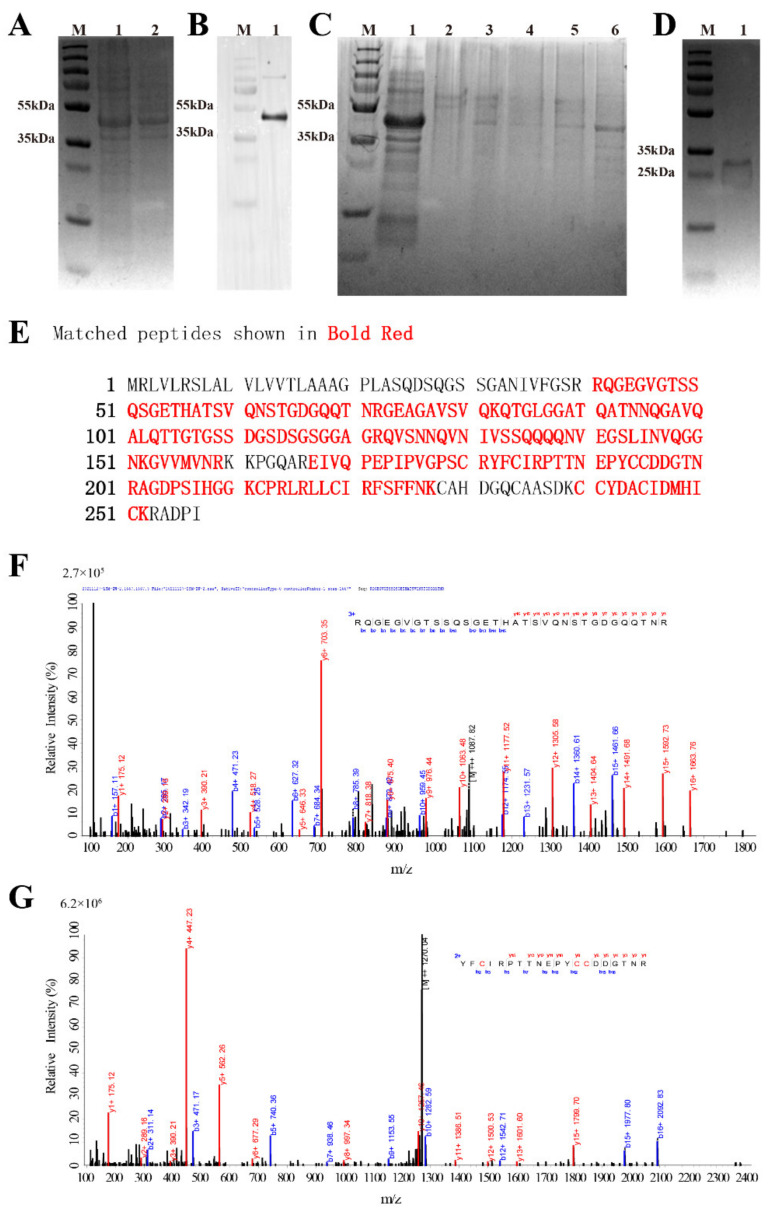
The acquisition process and MS spectrum analysis of *Aa*Crus1. (**A**) SDS-PAGE analysis of recombinant *Aa*Crus1(r*Aa*Crus1) expressed with a SUMO tag in *E. coli*. Lane M, protein marker; lane 1, total protein obtained from *E. coli* with IPTG induction; lane 2, total protein obtained from *E. coli* without induction. (**B**) Western Blot analysis of r*Aa*Crus1 expressed with a SUMO tag in *E. coli*. Lane M, protein marker; lane 1, total protein obtained from *E. coli* with IPTG induction. (**C**) r*Aa*Crus1 purified with nickel column chromatography. Lane M, protein marker; lane 1, protein not caught by the nickel column; lane 2, equilibration buffer; lane 3, eluent with 50 mM Imidazole; lane 4, eluent with 100 mM Imidazole, lane 5, eluent with 150 mM Imidazole; lane 6, eluent with 200 mM Imidazole. (**D**) SDS-PAGE analysis of r*Aa*Crus1 without SUMO tag. Lane M, protein marker; lane 1, r*Aa*Crus1 without SUMO tag. (**E**) Alignment of mass spectrometry results with r*Aa*Crus1 sequence. (**F**) MS spectrum of “RQGEGVGTSSQSGETHATSVQNSTGDGQQTNR”. (**G**) MS spectrum of “YFCIRPTTNEPYCCDDGTNR”.

As SDS-PAGE analysis (Figure 3A) showed no significant difference in the protein expressed by the bacteria before and after the induction of IPTG. Western Blot analysis (Figure 3B) was further performed. Western Blot analysis showed that there are obvious bands between 35 kDa and 55 kDa, which are in line with the size of His-SUMO-*Aa*Crus1 consisting of His-SUMO (~15 kDa) and *Aa*Crus1 (26.63 kDa). The 200 µM imidazole eluent can elute His-SUMO-*Aa*Crus1 from the Ni-column (Figure 3C, lane 6). After the treatment of SUMO enzyme, r*Aa*Crus1 without His-SUMO tag was obtained and characterized in SDS-PAGE at 25–35 kDa (Figure 3D).

### 3.3. LC-MS Identification Results

Using Mascot software to compare the amino acid sequence of r*Aa*Crus1, as shown in Figure 3E–G, a total of three peptides were detected, and the amino acid coverage reached 75%. It showed that with the prokaryotic expression obtained the fusion protein, the tag was successfully removed by the SUMO enzyme, and r*Aa*Crus1 without foreign amino acids was obtained.

### 3.4. Antimicrobial Activity of rAaCrus1

The antibacterial activity of r*Aa*Crus1 was tested against two Gram-positive and four *Vibrio* species, all of which were Gram-negative, by measuring the minimum inhibitory concentration (MIC) of each bacterium. As shown in Table 1, r*Aa*Crus1 exhibited apparent inhibitory activities against Gram-positive bacteria and against Gram-negative bacteria. For Gram-negative bacteria, r*Aa*Crus1 at a concentration of 7 μM can effectively inhibit the growth of *V. alginolyticus*. The most potent activity was detected against *V. parahemolyticus* and *V. harveyi*, with MIC of 28 μM. Compared with Gram-negative bacteria, *Aa*Crus1 has a lower MIC value for the two Gram-positive bacteria used in this study; both are 3.5 μM.

### 3.5. MD Simulations

**Figure 4:**
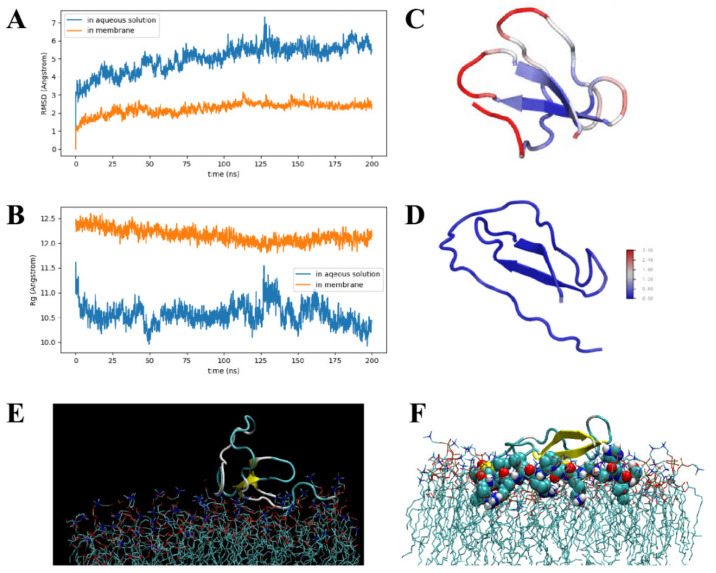
Molecular dynamics simulation. (**A**)The root–mean–square distances (RMSDs) of *Aa*Crus1 in aqueous solution and membrane. (**B**) The radii of gyration (Rg) of AaCrus1 in aqueous solution and membrane. (**C**) The root–mean–square fluctuations (RMSFs) of *Aa*Crus1 in aqueous solution. (**D**) The root–mean–square fluctuations (RMSFs) of *Aa*Crus1 in membrane. (**E**) *Aa*Crus1 and membrane binding simulation (initial stage). (**F**) *Aa*Crus1 and membrane binding simulation (medium stage).

The root–mean–square distances (RMSDs) and radii of gyration (Rg) of crustin in aqueous solution and membrane are plotted in Figure 4A,B. RMSDs reveal that both systems are already in equilibrium. Due to the membrane constraint, *Aa*Crus1 is less flexible than in aqueous solution. The mean Rg of *Aa*Crus1 in aqueous solution and membrane are 1.05 nm and 1.22 nm, respectively, indicating that in membrane, *Aa*Crus1 is more extended. In Figure 4C,D, the root–mean–square fluctuations (RMSFs) for both systems are given. In aqueous solution, while the beta sheet region is highly rigid, loop regions exhibit different flexibility. Loop Cys212-Leu218 is almost as rigid as the beta sheet region. The disulfide bond between Cys212 and Cys241 (2.04 Å) plays an important role in stabilizing this region. Loop Ser206-Cys212 is the most flexible part in *Aa*Crus1, and it exhibits the largest conformation change upon binding to the membrane. Loop Ser206-Phe222, deeply inserted into the membrane, anchors *Aa*Crus1 to the membrane (see Figure 4E,F); thus, it is probably an essential part in determining the antimicrobial function of *Aa*Crus1.

### 3.6. Microorganism-Binding Activity of rAaCrus1

**Figure 5:**
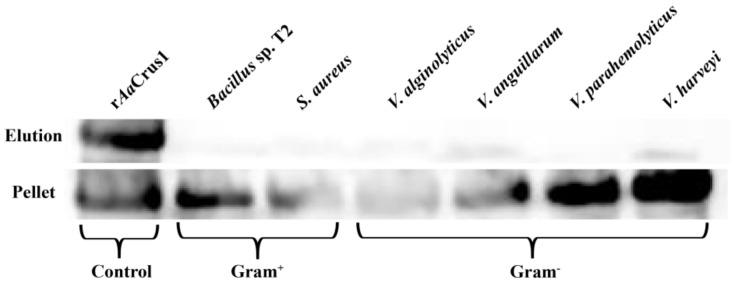
Bacterial agglutination and microorganism-binding activity of r*Aa*Crus1. r*Aa*Crus1 was detected by Western Blot assay after treatment with bacteria (Gram^+^ and Gram^−^). r*Aa*Crus1 was taken as a positive control. Up panel, elution fractions; bottom panel, final pellet fractions.

The results showed that r*Aa*Crus1 could strongly bind to two Gram-positive bacteria (*S. aureus*, *Bacillus* sp. T2) and four Gram-negative bacteria (*V. parahaemolyticus*, *V. harveyi*, *V. anguillarum*, *V. alginolyticus*). These results indicated that r*Aa*Crus1 might be binding to bacterial cells (Figure 5), which contribute to their antibacterial activity.

### 3.7. Agglutination Activity

After the agglutination test, r*Aa*Crus1 had a good agglutination effect on Gram-positive bacteria and Gram-negative bacteria. According to Table 2 and Supplementary Figure S2, we found that Ca^2+^ can increase the agglutination effect of *Aa*Crus1. The agglutination effect of r*Aa*Crus1 on Gram-positive bacteria is better than that on Gram-negative bacteria.

### 3.8. Effects of rAaCrus1 on the Bacterial Morphology

**Figure 6:**
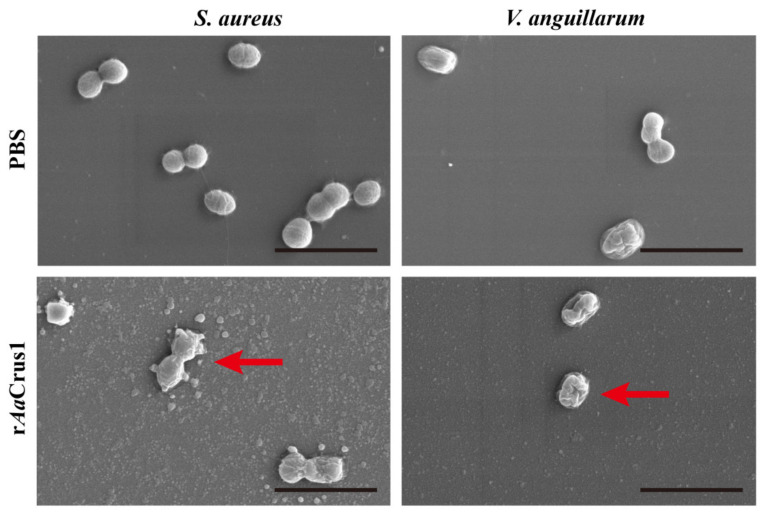
Morphological and cell membrane integrity changes of the bacterial cells treated with r*Aa*Crus1. Morphological changes of the bacterial cells treated with r*Aa*Crus1. About 10^6^ CFU·mL^−1^ bacteria were incubated with 2 × MIC of *Aa*Crus1 for 2 h and observed under scan electron microscopy. PBS were used as control. The scales are 3 μm. At the red arrow, the suspected bacterial content can be seen flowing out, and the cell surface shrinks.

After being treated with r*Aa*Crus1 for two hours, the surfaces of *S. aureus*, *V. anguillarum* and *V. alginolyticus* were uneven, the cells had severely shrunk and obvious cracks appeared on the surface of the cells to release the intracellular components (Figure 6 and Supplementary Figure S3).

### 3.9. Cell Membrane Permeability

Propidium iodide (PI) cannot pass through living cell membranes, but it can pass through damaged cell membranes to stain the nucleus, as we described in previous reports [39,40]. PI staining showed that after r*Aa*Crus1 treated *S. aureus*, *V. anguillarum* and *V. alginolyticus*, a large amount of PI was able to penetrate bacterial cells (Supplementary Figure S3), indicating that r*Aa*Crus1 could cause bacterial cell membrane destabilization.

### 3.10. Biofilm Inhibition

**Figure 7:**
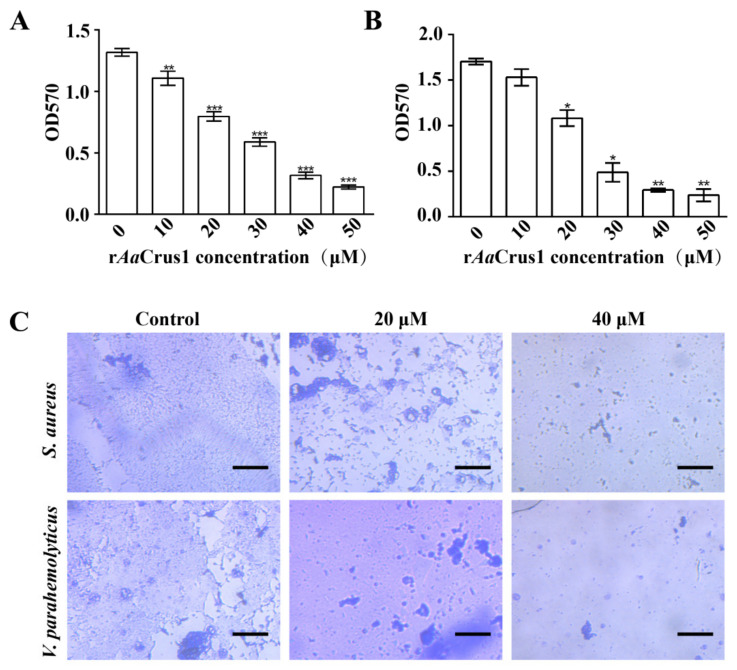
Biofilm inhibitory effect of *Aa*Crus1. Biofilm inhibitory concentration of r*Aa*Crus1 (0–50 μg/mL) on *S. aureus* (**A**) and *V. parahaemolyticus* (**B**). *, Compared with control (0 μM), *p* value < 0.01; **, Compared with control (0 μM), *p* value < 0.001; ***, Compared with control (0 μM), *p* value < 0.0001. (**C**) Control and treated with the different concentrations of r*Aa*Crus1 on *S. aureus* and *V. parahaemolyticus* biofilm development. The scales are 50 μm.

According to Figure 7, r*Aa*Crus1 disrupted development of *S. aureus* (Gram-positive bacteria) and *V. parahaemolyticus* (Gram-positive bacteria) biofilms. The higher the concentration of r*Aa*Crus1, the better the disrupting effect on the biofilm development.

## 4. Discussion

Crustins were first purified from the blood cells of the shore crab *C. maenas* and have a good antibacterial effect on Gram-positive bacteria. To date, a variety of crustins have been found in crustaceans such as amphipods, decapods and isopods. Generally, type I crustins are mainly active against Gram-positive bacteria [41,42]. Type II and type III crustins are active against both Gram-positive and Gram-negative bacteria [9,43]. Type IV crustins have protease inhibitory activity and antibacterial activity [44]. Most crustins with good antibacterial effect usually work at a concentration below 50 μM [41,42]. As non-decapod crustaceans, the habitat of *A. amphitrite* is more complex than decapod crustaceans such as crabs and prawns, suggesting that *A. amphitrite* encounter more various pathogenic microorganisms. It has served as an important source to discover new crustins.

After the construction and comparison of the protein database of crustins of *A. amphitrite*, we discovered an atypical crustin consisting of 257 amino acids, named *Aa*Crus1. Its molecular weight is 26.67 kDa, higher than the previously reported crustins. Despite having the same WAP domain as other crustins, *Aa*Crus1 additionally has a cysteine-rich region before the WAP domain. The predicted structure model shows that *Aa*Crus1, similar to other crustins [42], has a helix, a strand and some coils. There are 160 amino acids between the signal peptides and the cysteine-rich region of *Aa*Crus1, which exceeds the number of amino acids in this region of type I crustins listed in Figure 2A and Supplementary Table S2. There are 28 glycines between the signal peptide 1 and the cysteine-rich region of *Aa*Crus, however, BLAST and phylogenetic analysis showed that *Aa*Crus1 is closely related to *Pv*Crus (GenBank accession: QOL09947.1) and belongs to type I crustins. It is less than 50% similar to the decapod crustacean crustins (Supplementary Table S1). The molecular weight and sequences of *Aa*Crus1 are different from the reported crustins, possibly because *A. amphitrite* are not decapod crustaceans.

Given the discovery of *Aa*Crus1, a new crustin, we further applied a recombinant strategy to obtain a full sequence of the peptide. Compared with artificially synthesized AMPs [45], the prokaryotic recombinant expression system is low in cost and can form correctly folded proteins/peptides. Fusion expression AMPs with a SUMO tag can eliminate the toxicity of AMPs to prokaryotic hosts, prevent degradation by proteases and increase solubility [46,47]. The final AMPs were obtained by removing the SUMO tag by enzymatic digestion and purification. SDS-PAGE and LC-MS characterisation of the purified AMPs further confirmed the recombinant products, r*Aa*Crus1. The missing region from Cys228 to Lys239 under LC-MS analysis may be caused by the highly constructed four-disulfide bond for difficulty of enzymatic digestion. MD simulations shown that disulfide bonds between Cys212 and Cys241 play an important role in stabilizing this region. These results indicate that the structure of *Aa*Crus1 assembled by the prokaryotic expression system is consistent with the prediction.

Based on the results towards multiple *Vibrio* pathogens commonly existing in the aquaculture industry, our antibacterial activity result showed that r*Aa*Crus1 is similar to other crustins and has broad-spectrum antibacterial activity against Gram-positive and Gram-negative bacteria. Bacterial agglutination test, microbial binding test, SEM and PI staining results indicate that r*Aa*Crus1 may achieve antibacterial status by agglutinating bacteria and damaging cell membranes.

Agglutinating bacteria is one of the ways in which AMPs work. Positively charged AMPs can neutralize the charge of certain surface components (lipopolysaccharide, lipoteichoic acid or peptidoglycan) of bacteria, reducing the electrostatic repulsion between bacteria, leading to agglutination [22,48]. The positively charged r*Aa*Crus1 (with lysine and arginine) can neutralize the anions on the surface of the bacteria and reduce the electrostatic repulsion on the surface of the bacteria. The binding of r*Aa*Crus1 to bacteria was detected by Western Blot technology. It was found that r*Aa*Crus1 can bind strongly to the tested bacteria; r*Aa*Crus1 may bind to one or more bacterial surface components and interact with the bacterial surface. This may be the reason why r*Aa*Crus1 can agglutinate two kinds of Gram-positive bacteria and four kinds of Gram-negative bacteria.

Some AMPs can exert antibacterial effects across the bacterial plasma membrane with the help of lipoteichoic acid and wall teichoic acid on the bacterial surface [49,50]. The peptidoglycan on the bacterial membrane can attract cationic AMPs to the bacterial cell membrane and promote insertion into the membrane, which will eventually lead to membrane leakage [51,52]. Our MD simulations showed that the coil part of the *Aa*Crus1 structure first binds tightly to the phosphate surface of the upper layer of the bacterial cell membrane, and then Ser206-Phe222 in the WAP domain binds to the cell membrane and deeply inserts into it and anchors it. This may be one of the reasons for the strong binding of r*Aa*Crus1 and bacteria in the microbial binding experiment. Electron microscopy further showed that the bacteria treated with r*Aa*Crus1 showed cell surface atrophy and the contents flowed out, indicating that the bacterial cell membrane was damaged. The results of PI staining showed that r*Aa*Crus1 changed the permeability of the bacterial cell plasma membrane. Therefore, we believe that the antibacterial mode of action of *Aa*Crus1 may be that its WAP domain and glycine-rich region both play a role in binding to bacteria. The WAP domain and the cationic amino acid residues in it are deeply embedded in the cell membrane and cause the bacterial membrane to penetrate. Finally, *Aa*Crus1 crosses to the bacterial cell plasma membrane, and the WAP domain continues to function to kill the bacteria.

Bacteria in the biofilm are a thousand times more resistant to antibiotics than scattered bacteria [53]. Some AMPs can inhibit the development of biofilms by degrading or preventing the production of important biofilm matrix components [54]. It is also possible to prevent the initial attachment to the surface by inhibiting swimming or swarm movement or interfering with flagella assembly to inhibit the development of biofilms. Through crystal violet staining, we found that *Aa*Crus1 can inhibit the development of biofilms. Like other crustins, *Aa*Crus1 may have protease inhibitory activity and affect the production of biofilm matrix components.

## 5. Conclusions

In this study, we discovered and characterized a new crustin, *Aa*Crus1 (recombinant version r*Aa*Crus1). By using makeblastdb for build a local database of crustins, retrieving crustins from barnacle genomes using blastp and the local crustins database, we found new non-decapod crustacean crustin. The recombinant expression further assisted us to obtain r*Aa*Crus1 with His-SUMO tag, followed by the His-SUMO tag removal. All the recombinant analogues were characterized by SDS-PAGE, Western Blot and LC-MS. The bioassay showed its potent and broad-spectrum antibacterial activity and bacterial agglutination activity. Additional mechanistic study and computational modelling further suggested its possible antibacterial mechanism. *Aa*Crus1 can help organisms resist microbial attack by agglutinating cells and can also bind to bacterial surfaces and disrupt bacterial cell membranes, ultimately leading to bacterial death. Our results suggest that this new *Aa*Crus1 can serve as a template for the further development and design of promising antimicrobial agents and their applications in aquaculture.

## Figures and Tables

**Table 1:** The minimal inhibitory concentration (MIC) of *Aa*Crus1 and ampicillin against Gram-positive and Gram-negative bacteria.

Microorganism	Minimal Inhibitory Concentrations
	r*Aa*Crus1
**Gram^+^**	
*S. aureus*	3.5 μM
*Bacillus* sp. T2	3.5 μM
**Gram^−^**	
*V. alginolyticus*	7 μM
*V. parahemolyticus*	28 μM
*V. harveyi*	28 μM
*V. anguillarum*	14 μM

**Table 2:** Agglutinating activity of *Aa*Crus1. Minimal agglutinating concentration is defined as the lowest protein concentration obtained with obvious agglutination, compared with the negative control.

Microorganisms	Minimal Agglutinating Concentrations (μM)	
	r*Aa*Crus1 + Ca^2+^	r*Aa*Crus1
**Gram^+^**		
*S. aureus*	0.875	3.5
*Bacillus* sp. T2	1.75	3.5
**Gram^−^**		
*V. alginolyticus*	3.5	14
*V. parahemolyticus*	1.75	7
*V. harveyi*	3.5	7
*V. anguillarum*	7	14

## Data Availability

All data available are reported in the article.

## Supplementary Materials

The following are available online at https://www.mdpi.com/article/10.3390/pharmaceutics14020413/s1, Table S1: Blast result of *Aa*Crus1 with other crustins in NCBI. Table S2: Information on the crustins used in the sequence alignment of Figure 2A. Table S3: Information on the crustin-I used to construct the evolutionary tree. Table S4: Information on the crustin-II used to construct the evolutionary tree. Table S5: Information on the crustin-III used to construct the evolutionary tree. Table S6: MIC of r*Aa*Crus1 with tag and r*Aa*Crus1. Figure S1: pSmartI-SUMO-*Aa*Crus1 map. Figure S2: Agglutination of bacteria (Gram^+^ and Gram^−^) induced by r*Aa*Crus1. Figure S3: The effect of Aarus1 on bacterial cell membrane integrity. Supplementary data: The nucleotide sequence of codon-optimized *Aa*Crus1.
