# Supplementary material for: Discovery and Characterization of a New Crustin Antimicrobial Peptide from Amphibalanus amphitrite
Source: Pharmaceutics. 2022 Feb 14;14(2):413. doi: 10.3390/pharmaceutics14020413 (PMC8877177; doi:10.3390/pharmaceutics14020413)
Supplement: Supplementary file 1 [file pharmaceutics-14-00413-s001.zip › pharmaceutics-1575355-supplementary.pdf]

pharmaceutics

Discovery and Characterization of a New Crustin Antimicrobial Peptide from *Amphibalanus amphitrite*

Wei Zhang, Xiaohang Xu, Jun Zhang, Ting Ye, Qiao Zhou, Ying Xu, Wenyi Li, Zhangli Hu and Chenjing Shang

Table S1. Blast result of *Aa*Crus1 with other crustins in NCBI.

Description	Scientific Name	Total Score	Query Cover	Per. ident	Acc. Len	Accession
hypothetical protein FJT64_024809 [*Amphibalanus amphitrite*]	*Amphibalanus amphitrite*	520	100%	100	257	KAF0303230.1
type Ia crustin cruIa-6 [*Penaeus vannamei*]	*Penaeus vannamei*	68.6	28%	48.1	108	QOL09947.1
antileukoproteinase-like [*Penaeus vannamei*]	*Penaeus vannamei*	68.6	28%	48.1	122	XP_027219905.1
calcium-binding protein P-like [*Penaeus monodon*]	*Penaeus monodon*	65.9	29%	46.99	169	XP_037792256.1
type I crustin 6 [*Scylla paramamosain*]	*Scylla paramamosain*	66.6	30%	46.59	113	AUV47158.1
carcinin [*Portunus pelagicus*]	*Portunus pelagicus*	60.5	31%	46.59	108	ABM65762.1
antileukoproteinase-like [*Penaeus vannamei*]	*Penaeus vannamei*	67.4	30%	46.43	120	XP_027219667.1
antileukoproteinase-like [*Homarus americanus*]	*Homarus americanus*	70.5	29%	46.15	151	XP_042240247.1
41 kDa spicule matrix protein-like [*Penaeus vannamei*]	*Penaeus vannamei*	65.5	29%	45.78	176	XP_027227947.1
type IIa crustin cruIIa-7 [*Penaeus vannamei*]	*Penaeus vannamei*	65.1	29%	45.78	176	QOL09961.1

crustin I-2 [*Penaeus japonicus*]	*Penaeus japonicus*	67.4	29%	45.45	124	UGN74322.1
type I crustin 109 [*Rimicaris* sp.]	*Rimicaris* sp.	60.1	29%	45	109	QXF28499.1
CruI-1 [*Penaeus japonicus*]	*Penaeus japonicus*	64.3	31%	43.62	110	AME17866.1
type Ib crustin cruIb-1 [*Penaeus chinensis*]	*Penaeus chinensis*	63.9	29%	43.59	126	QOL09976.1
putative crustin-like antimicrobial peptide 17 [*Homarus americanus*]	*Homarus americanus*	60.5	28%	43.59	116	KAG7172931.1
galectin-3-like [*Penaeus japonicus*]	*Penaeus japonicus*	60.5	29%	43.37	165	XP_042892210.1
androgenic gland-specific protein [*Macrobrachium rosenbergii*]	*Macrobrachium rosenbergii*	65.9	35%	42.55	111	ACL15396.1
perlwapin-like [*Penaeus japonicus*]	*Penaeus japonicus*	62	31%	42.55	110	XP_042892931.1
crustin 3 [*Portunus trituberculatus*]	*Portunus trituberculatus*	64.3	31%	42.39	115	AFU61584.1
crustin 3 [*Portunus trituberculatus*]	*Portunus trituberculatus*	63.2	31%	42.39	115	AFU61580.1
waprin-Phi1-like [*Portunus trituberculatus*]	*Portunus trituberculatus*	62.4	31%	42.39	115	XP_045139053.1
crustin 3 [*Portunus trituberculatus*]	*Portunus trituberculatus*	62.4	31%	42.39	115	AFU61579.1
waprin-Phi1-like [*Portunus trituberculatus*]	*Portunus trituberculatus*	62.4	31%	42.39	115	XP_045139055.1
waprin-Phi1-like [*Portunus trituberculatus*]	*Portunus trituberculatus*	62	31%	42.39	115	XP_045139054.1
type IIa crustin cruIIa-8 [*Penaeus vannamei*]	*Penaeus vannamei*	67.4	28%	42.31	156	QOL09962.1
whey acidic protein-like [*Amphibalanus amphitrite*]	*Amphibalanus amphitrite*	57.4	29%	42.11	119	XP_043240188.1
acanthoscurrin-2-like [*Portunus trituberculatus*]	*Portunus trituberculatus*	62.8	31%	41.57	242	XP_045136931.1

acanthoscurrin-2-like [*Penaeus monodon*]	*Penaeus monodon*	68.2	29%	41.56	178	XP_037792050.1
keratin-associated protein 5-5-like [*Penaeus japonicus*]	*Penaeus japonicus*	66.2	29%	41.56	172	XP_042868296.1
keratin-associated protein 5-4-like isoform X1 [*Homarus americanus*]	*Homarus americanus*	63.9	28%	41.56	191	XP_042203450.1
putative crustin-like antimicrobial peptide 8 [*Homarus americanus*]	*Homarus americanus*	60.5	28%	41.56	140	KAG7156562.1
crustin 114 [*Rimicaris* sp.]	*Rimicaris* sp.	61.2	29%	41.46	114	UCR17161.1
crustin-like protein 1 [*Mytilus coruscus*]	*Mytilus coruscus*	60.8	30%	41.18	184	QXT26515.1
crustin 164 [*Rimicaris* sp.]	*Rimicaris* sp.	60.5	30%	40.96	164	UCR17160.1
antimicrobial peptide type 1 precursor Ic [*Pandalus japonicus*]	*Pandalus japonicus*	61.2	28%	40.79	117	AGU01540.1
glycine-rich cell wall structural protein-like [*Penaeus* monodon]	*Penaeus monodon*	69.7	30%	40.74	437	XP_037792051.1
WAP-type 'four-disulfide core' domain [*Trinorchestia longiramus*]	*Trinorchestia longiramus*	127	28%	40.54	394	KAF2367915.1
ATP-dependent RNA helicase glh-2-like [*Penaeus vannamei*]	*Penaeus vannamei*	63.2	25%	40.3	154	XP_027215341.1

Table S2. Information on the crustins used in the sequence alignment of Figure 2A.

Abbreviation	Explanation	NCBI Accession Number
PvCrus_QQL09947.1	*Penaeus vannamei* type Ia crustin cruIa-6	QQL09947.1
PtCrus_ACM89167.2	*Portunus trituberculatus* crustin antimicrobial peptide	ACM89167.2
SsCrus_ADW11096.1	*Scylla serrata* crustin	ADW11096.1
Carcincin_CAD20734.1	*Carcinus maenas* carcinin	CAD20734.1
HaCrus_ACJ06763.1	*Hyas araneus* crustin Ha1	ACJ06763.1
PtCrus_AFU61580.1	*Portunus trituberculatus* crustin 3	AFU61580.1
SpCrus_AUV47158.1	*Scylla paramamosain* type I crustin 6	AUV47158.1
HaCrus_ABM92333.1	*Homarus americanus* crustin-like protein precursor	ABM92333.1
PjCrus_AFN80342.1	*Pandalus japonicus* antimicrobial peptide type 1 precursor	AFN80342.1

Table S3. Information on the crustin-I used to construct the evolutionary tree.

Type	Abbreviation	Explanation	NCBI Accession Number
I	PvCrusQQL09947.1	*Penaeus vannamei* type Ia crustin cruIa-6	QQL09947.1
	PtCrusACM89167.2	*Portunus trituberculatus* crustin antimicrobial peptide	ACM89167.2
	SsCrusADW11096.1	*Scylla serrata* crustin	ADW11096.1
	CarcincinCAD20734.1	*Carcinus maenas* carcinin	CAD20734.1
	HaCrusACJ06763.1	*Hyas araneus* crustin Ha1	ACJ06763.1
	PtCrusAFU61580.1	*Portunus trituberculatus* crustin 3	AFU61580.1
	SpCrusAUV47158.1	*Scylla paramamosain* type I crustin 6	AUV47158.1
	HaCrusABM92333.1	*Homarus americanus* crustin-like protein precursor	ABM92333.1
	PjCrusAFN80342.1	*Pandalus japonicus* antimicrobial peptide type 1 precursor	AFN80342.1

Table S4. Information on the crustin-II used to construct the evolutionary tree.

**Type**	**Abbreviation**	**Explanation**	**NCBI Accession Number**
II	ReCrusQIE09215.1	*Rimicaris exoculata* re-crustin typeII	QIE09215.1
	PjCrus1 ACU25382.1	*Panulirus japonicus* typeII crustin	ACU25382.1
	PmCrusACQ66004.1	*Penaeus monodon* typeII crustin	ACQ66004.1
	PcCrusAAX63903.1	*Penaeus chinensis* typeII crustin	AAX63903.1
	PpCrusABM63361.1	*Penaeus paulensis* typeII crustin	ABM63361.1
	PsCrusABO93323.1	*Penaeus subtilis* typeII crustin	ABO93323.1
	PvCrusIIAAS59739.1	*Penaeus vannamei* typeII crustin	AAS59739.1
	PbCrusABQ96197.1	*Penaeus brasiliensis* typeII crustin	ABQ96197.1

Table S5. Information on the crustin-III used to construct the evolutionary tree.

Type	Abbreviation	Explanation	NCBI Accession Number
III	PmSWD3ACF28466.1	*Penaeus monodon* single whey acidic protein domain-containing protein isoform 3	ACF28466.1
	PvSWDAAS17722.1	*Penaeus vannamei* single WAP domain protein	AAS17722.1
	PmSWDACF28465.1	*Penaeus monodon* single whey acidic protein domain-containing protein isoform 2	ACF28465.1
	PcSWDABN09668.1	*Penaeus chinensis* single whey acidic protein domain-containing peptide	ABN09668.1
	PvSWDAAS38513.1	*Penaeus vannamei* single WAP domain-containing protein	AAS38513.1

Table S6. MIC of r*Aa*Crus1 with tag and r*Aa*Crus1

**Microorganisms**	**Minimal inhibitory concentrations (μM)**	
	**r*Aa*Crus1**	**r*Aa*Crus1 with tag**
	**Gram^{+}**	
S.aureus	3.5 μM	3.5 μM
	**Gram^{-}**	
V.parahemolyticus	28 μM	28 μM

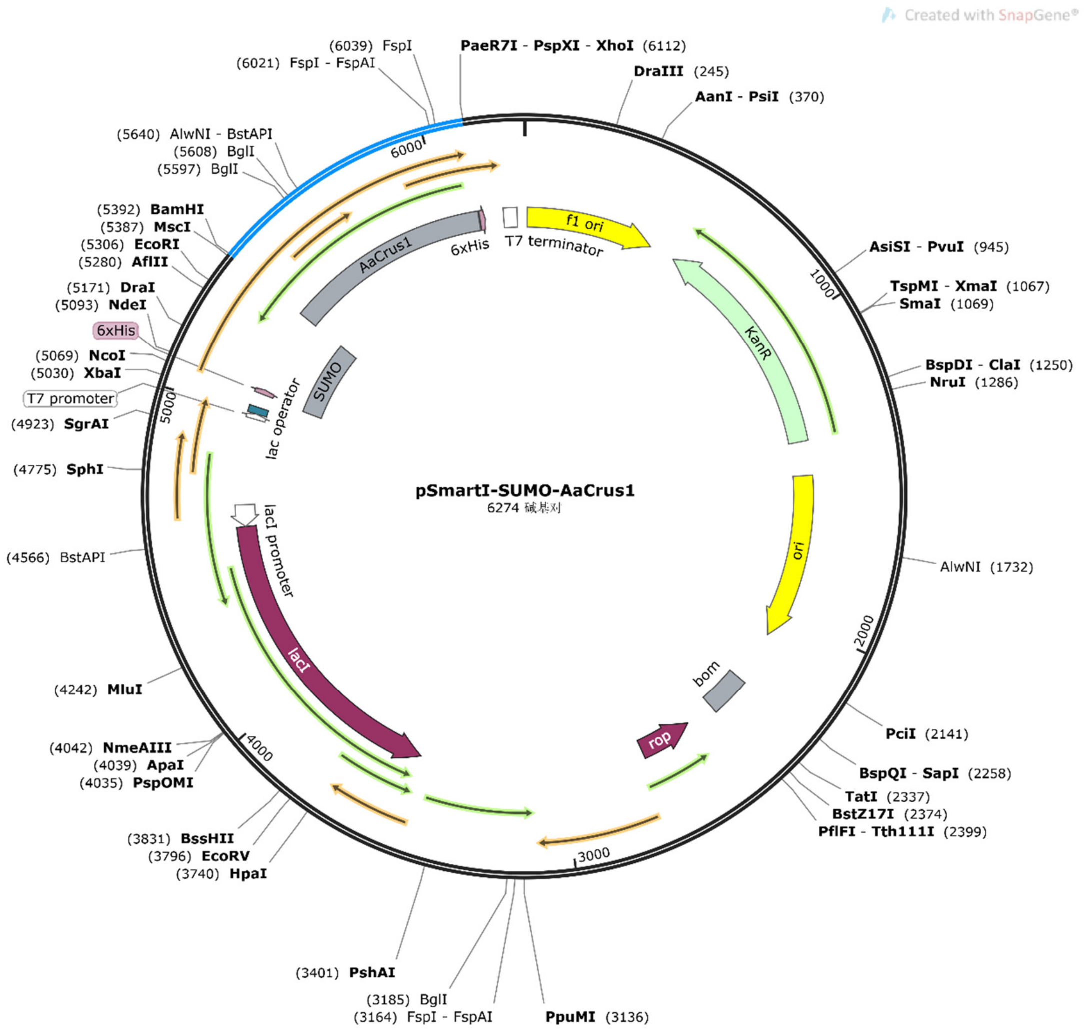

Figure S1. pSmartI-SUMO-*Aa*Crus1 map

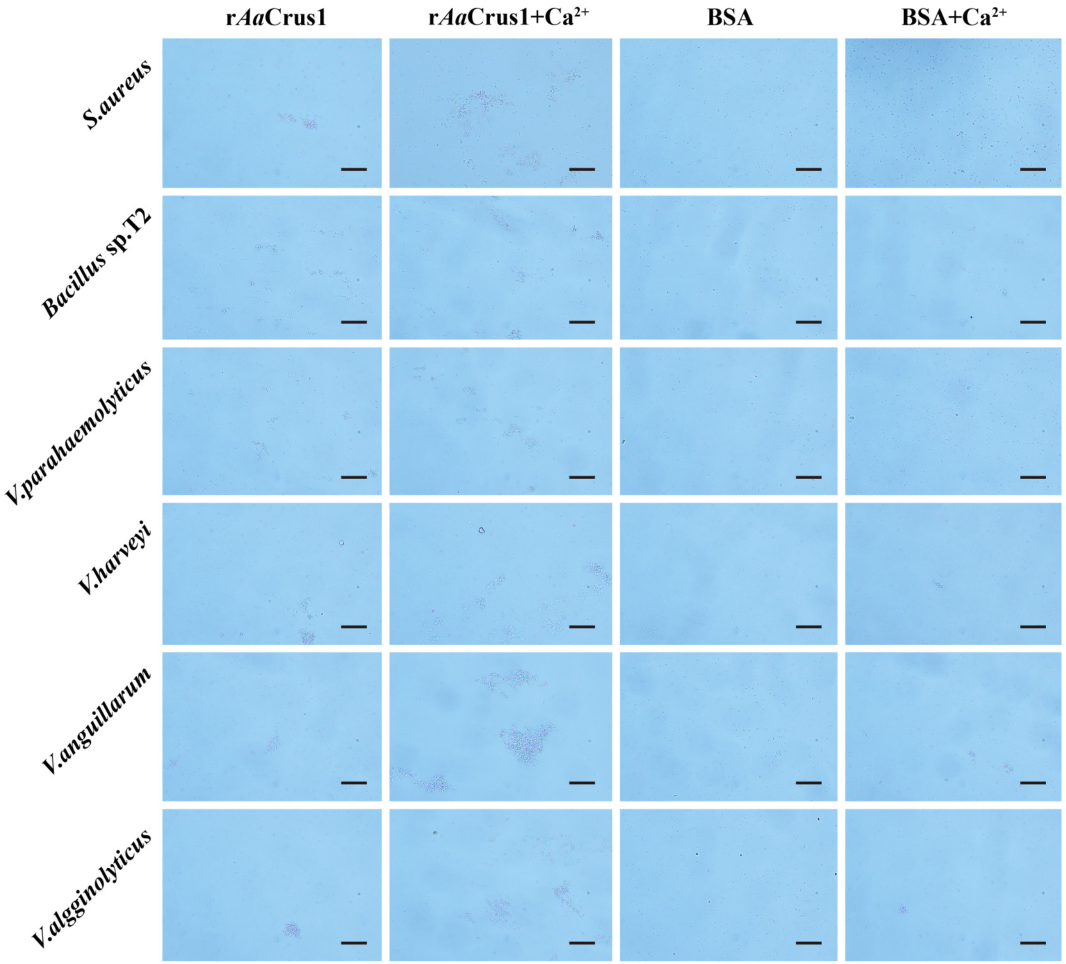

Figure S2. Agglutination of bacteria (Gram$^+$ and Gram$^-$) induced by r*Aa*Crus1. About 1 × 10^8 CFU·mL^{-1} bacteria were incubated with MAC of r*Aa*Crus1 for 1 h and observed under optical microscope. BSA and BSA with 5 mM $CaCl_2$ were used as control. The scales are 50 μm.

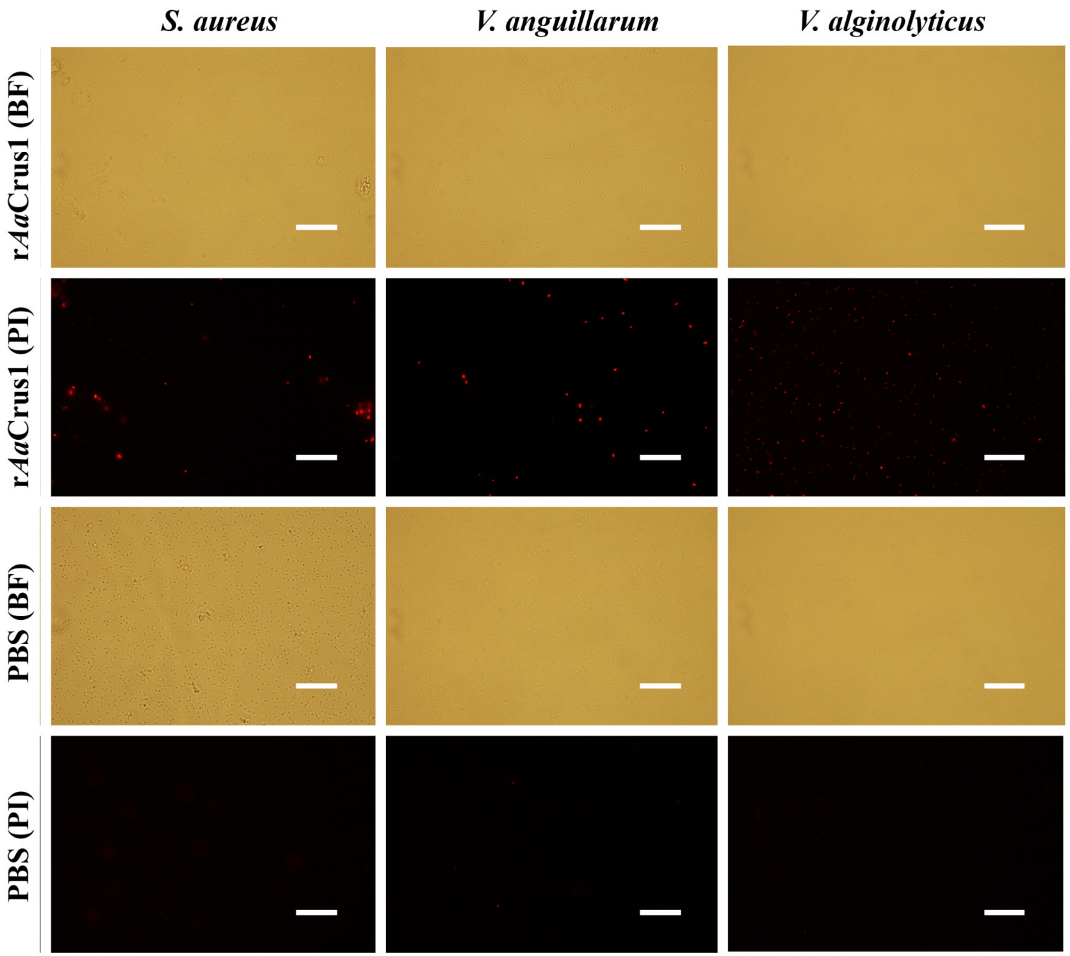

Figure S3. The effect of *Aa*rus1 on bacterial cell membrane integrity. About 1 × 10^6 CFU·mL^{-1} bacteria were incubated with 2×MIC of r*Aa*Crus1 for 2 h. PI, The cells were stained with PI and observed for PI uptake with a fluorescence microscope; BF, The bright field image.The scales are 50 μm.

Supplement data. The nucleotide sequence of codon-optimized *Aa*Crus.

GGATCCCCTCTGGCAAGCCAGGATAGTCAGGGCAGTAGTGGCGCAAATATTGTGTTTGGCAGTCGCCGTCAGGGTGAAGGTG

TGGGTACCAGTAGTCAGAGTGGTGAAACCCATGCAACCAGCGTTCAGAATAGTACCGGTGACGGCCAGCAGACCAATCGCG

GTGAAGCAGGTGCAGTTAGTGTTCAGAAACAGACCGGCCTGGGCGGCGCCACCCAGGCAACCAATAATCAGGGCGCAGTGC

AGGCACTGCAGACCACCGGCACCGGTAGCAGCGATGGTAGTGATAGTGGTAGTGGCGGCGCAGGTCGTCAGGTTAGTAATAA

TCAGGTTAATATTGTGAGCAGCCAGCAGCAGCAGAATGTGGAAGGTAGCCTGATTAATGTGCAGGGCGGCAATAAGGGCGTT

GTTATGGTGAATCGTAAAAAACCGGGTCAGGCCCGTGAAATTGTTCAGCCGGAACCGATTCCGGTGGGTCCGAGCTGCCGTTA

TTTTTGCATTCGTCCGACCACCAATGAACCGTATTGCTGCGATGATGGCACCAATCGTGCCGGTGACCCGAGCATTCATGGTG

GCAAATGCCCGCGTCTGCGTCTGCTGTGCATTCGCTTTAGTTTCTTTAATAAGTGCGCACATGATGGTCAGTGCGCAGCCAGTG

ATAAATGCTGCTATGATGCCTGTATTGATATGCATATTTGCAAACGCGCAGATCCGATTTAACTCGAG
